# Activation of Frizzled-7 attenuates blood–brain barrier disruption through Dvl/β-catenin/WISP1 signaling pathway after intracerebral hemorrhage in mice

**DOI:** 10.1186/s12987-021-00278-9

**Published:** 2021-09-26

**Authors:** Wei He, Qin Lu, Prativa Sherchan, Lei Huang, Xin Hu, John H. Zhang, Haibin Dai, Jiping Tang

**Affiliations:** 1 Department of Pharmacy, Second Affiliated Hospital, Zhejiang University School of Medicine, Hangzhou, 310009 Zhejiang China; 2 Department of Physiology and Pharmacology, Center for Neuroscience Research, Loma Linda University School of Medicine, Loma Linda, CA 92354 USA; 3 Department of Neurosurgery, Sir Run Run Shaw Hospital, Zhejiang University School of Medicine, Hangzhou, Zhejiang China; 4 Department of Neurosurgery, Loma Linda University School of Medicine, Loma Linda, CA 92354 USA; 5 Department of Neurosurgery, West China Hospital, Sichuan University, Chengdu, Sichuan China; 6 Department of Anesthesiology, Loma Linda University School of Medicine, Loma Linda, CA 92354 USA; 7 Department of Physiology and Pharmacology, School of Medicine, Loma Linda University, 11041 Campus Street, Loma Linda, CA 92354 USA

**Keywords:** Intracerebral hemorrhage, Blood–brain barrier, Frizzled-7, Dvl, β-Catenin, WISP1

## Abstract

**Background:**

Destruction of blood–brain barrier (BBB) ​​is one of the main mechanisms of secondary brain injury following intracerebral hemorrhage (ICH). Frizzled-7 is a key protein expressed on the surface of endothelial cells that controls vascular permeability through the Wnt-canonical pathway involving WNT1-inducible signaling pathway protein 1 (WISPI). This study aimed to investigate the role of Frizzled-7 signaling in BBB preservation after ICH in mice.

**Methods:**

Adult CD1 mice were subjected to sham surgery or collagenase-induced ICH. Frizzled-7 activation or knockdown was performed by administration of Clustered Regularly Interspaced Palindromic Repeats (CRISPR) by intracerebroventricular injection at 48 h before ICH induction. WISP1 activation or WISP1 knockdown was performed to evaluate the underlying signaling pathway. Post-ICH assessments included neurobehavior, brain edema, BBB permeability, hemoglobin level, western blot and immunofluorescence.

**Results:**

The brain expressions of Frizzled-7 and WISP1 significantly increased post-ICH. Frizzled-7 was expressed in endothelial cells, astrocytes, and neurons after ICH. Activation of Frizzled-7 significantly improved neurological function, reduced brain water content and attenuated BBB permeability to large molecular weight substances after ICH. Whereas, knockdown of Frizzled-7 worsened neurological function and brain edema after ICH. Activation of Frizzled-7 significantly increased the expressions of Dvl, β-Catenin, WISP1, VE-Cadherin, Claudin-5, ZO-1 and reduced the expression of phospho-β-Catenin. WISP1 knockdown abolished the effects of Frizzled-7 activation on the expressions of VE-Cadherin, Claudin-5 and ZO-1 at 24 h after ICH.

**Conclusions:**

Frizzled-7 activation potentially attenuated BBB permeability and improved neurological deficits after ICH through Dvl​​/β-Catenin/WISP1 pathway. Frizzled-7 may be a potential target for the development of ICH therapeutic drugs.

**Supplementary Information:**

The online version contains supplementary material available at 10.1186/s12987-021-00278-9.

## Introduction

Intracerebral hemorrhage (ICH) is a life-threatening neurological disorder with high mortality (the 5-year mortality rate higher than 50%) [1, 2]. There is no proven effective medical or surgical treatments available for ICH patients, although surgical decompression for cerebellar hemorrhages is accepted as potentially life-saving approach [1]. Increasing evidence has shown that blood–brain barrier (BBB) dysfunction is one of the key mechanisms of brain injury following ICH and closely related to poor outcomes [1, 3]. The increased BBB permeability contributes to vascular-derived brain edema after ICH [2, 4]. Hematoma promotes capillary leakage and breaks the integrity of endothelium tight junction, after which the mass effect of the exudate causes delayed neurological deterioration [5]. Tight junction proteins (TJPs), like claudins, occludin and junction adhesion molecule-1, span the membrane of two adjacent endothelial cells of the BBB, which forms a protective barrier between the brain and blood. The degradation of these transmembrane proteins reduces the barrier integrity and increases paracellular permeability thereby worsening brain edema [6, 7]. Therefore, treatment strategies against BBB disruption would help attenuate secondary brain damage after ICH.

Frizzled family receptor 7 (FZD7) is one of the FZD receptors that mediate the classical and non-classical Wnt signaling [8]. It is a new receptor identified on the surface of endothelial cells in the peripheral [9] and retinal vasculature [10]. The Human Protein Atlas database shows that FZD7 is expressed in the human and mouse brain. Additionally, the Barres brain RNA-Seq database and the Betzholtz single-cell RNA-Seq database show that FZD7 had varying levels of gene expression in astrocytes, pericytes, neurons and endothelial cells in the mouse brain. However, the function of FZD7 in cerebral blood vessels needs to be further elucidated. FZD7 controls vascular permeability through Wnt canonical pathway and activates endothelial cell signaling by recruiting disheveled (Dvl) to the cell membrane [9, 10]. FZD7 knockdown or depletion in mice resulted in the disruption of adherens junction and increased peripheral vascular permeability in vivo and in vitro [9]. In a study of the gut barrier function, inactivation of Wnt signaling by down-regulating FZD7 expression significantly reduced TJ proteins zona occludens-1 (ZO-1) and Claudin-1 [11]. Furthermore, the activation of Wnt/β-catenin signaling has been shown to maintain the integrity of brain microcirculation and control BBB permeability [12]. Activated nuclear β-catenin can induce the transcription of WNT1-inducible signaling pathway protein 1 (WISP1/CCN4), which is a downstream effector of Wnt/β-catenin signaling [13]. In melanoma cells, WISP1 knockout down regulated the mesenchymal marker N-cadherin and fibronectin [13]. However, the effects of FZD7 on BBB integrity and its signaling pathway after ICH have not been studied. Furthermore, whether WISP1 as a downstream molecule is involved in FZD7 mediated BBB protection has not been explored.

The aim of our study was to evaluate the potential effect of FZD7 in BBB protection after ICH. We hypothesized that FZD7 activation would potentially contribute to maintain BBB integrity following ICH and this protection may be mediated through BBB junction protection via Dvl/β-Catenin/WISP1 signaling pathway.

## Methods

### Animals

A total of 262 adult male CD1 mice weighing 25–35 g (Charles River, MA, USA) were used. Mice were reared with a standard 12 h light/12 h dark cycle in a controlled temperature and humidity room with unlimited supply of water and food. All animal experiment procedures were approved by the Institutional Animal Care and Use Committee of Loma Linda University and were carried out in accordance with the Guide for the Care and Use of Laboratory Animals developed by the National Institutes of Health.

### ICH model

We induced the ICH model as previously described [14, 15]. Briefly, the mice were intraperitoneally injected with ketamine/xylazine (100/10 mg/kg), and were placed prone in a stereotactic head frame (Kopf Instruments, Tujunga, CA) after being deeply anesthetized. A cranial burr hole (1 mm) was drilled and a 27-gauge needle was inserted into the right basal ganglia (coordinates: 1.5 mm lateral to the midline, 0.9 mm posterior to the bregma, 4 mm below the dura mater). Collagenase (0.075 U dissolved in 0.5 µL saline, VII-S; Sigma Aldrich, St Louis, MO) was injected with a micro infusion pump (Harvard Instruments, Holliston, MA) at a rate of 0.25 µL/min. Sham mice were subjected to the identical surgical procedures but were injected with 0.5 µL saline and not the ICH-inducing collagenase. After the end of injection, the needle was left for another 10 min to prevent extravasation of collagenase solution. The burr hole was sealed with bone wax after which the needle was pulled out and the skin incision was sutured, and the mouse was allowed to recover. The body temperature of the mice was maintained with a heating blanket (37 ± 0.5 °C) controlled by an electronic thermostat throughout the procedure.

### Administration of CRISPR

Each mouse received a total of 1 µg (2 µL) of Clustered Regularly Interspaced Palindromic Repeats (CRISPR) which was administered by intracerebroventricular injection at 48 h prior to ICH induction. The CRISPR was injected at a rate of 0.67 µL/min at the following coordinates: 1.0 mm lateral and 0.2 mm posterior to the bregma, 2.3 mm below the dura mater. Frizzled-7 CRISPR/Cas9 Knockout (KO) Plasmid (sc-420437, Santa Cruz Biotechnology, USA) and Frizzled-7 CRISPR Activation Plasmid (sc-420437-ACT, Santa Cruz Biotechnology, USA) were used to knockdown and activate the expression of FZD7, respectively. WISP1 CRISPR/Cas9 KO Plasmid (sc-423705, Santa Cruz Biotechnology, USA) and WISP1 CRISPR Activation Plasmid (sc-423705-ACT, Santa Cruz Biotechnology, USA) were used to knockdown and activate the expression of WISP1, respectively. Control CRISPR/Cas 9 Plasmid (sc-418922, Santa Cruz Biotechnology, USA) and Control CRISPR Activation Plasmid (sc-437275, Santa Cruz Biotechnology, USA) were administered as negative controls for CRISPR/Cas9 KO Plasmid and CRISPR Activation Plasmid, respectively.

### Neurobehavioral tests

Neurobehavioral functions were evaluated by an investigator blinded to the groups. Neurobehavioral scores were assessed by an investigator blinded to group information. Short-term assessments included the modified Garcia test, limb placement test, and corner turn test as described previously [16]. Long-term assessments included rotarod test, foot fault test, and Morris water maze test as previously shown [17].

### Brain water content

The wet/dry weight method was used to measure brain water content at 24 and 72 h post-ICH [18]. The mouse brain was separated into five parts: cerebellum (internal control), ipsilateral and contralateral cortex, and ipsilateral and contralateral basal ganglia. The wet weight (WW) of brain specimen was obtained by an electronic analytical balance (APX-60, Denver Instrument, NY), and the dry weight (DW) was obtained after the brain samples were dried in an oven at 100 °C for 72 h. The formula for calculating the percentage of brain water content was: [(WW − DW/WW] × 100%.

### BBB permeability

The BBB permeability was evaluated by Evans blue extravasation as previously described [3]. The Evans blue dye was prepared into a 4% solution with normal saline and allowed to circulate in the mice for 3 h after intraperitoneal injeciton (4 mL/kg). After that, the mice were deeply anesthetized and perfused through the heart with 50 mL of ice-cold phosphate buffered saline (PBS). The brains were taken out, separated into left and right halves, and then stored in − 80 °C freezer until analysis. Each brain specimen was added with 1100 µL PBS, homogenized, sonicated and centrifuged (4 °C, 14,000 rcf, 30 min) to obtain the supernatant. The same amount of trichloroacetic acid (TCA) was added and centrifuged again (4 °C, 14,000 rcf, 30 min) to obtain the supernatant. The concentration of Evans blue was measured at 610 nm with a spectrophotometer (Thermo Fisher, MA) and quantified according to the standard curve.

### Hematoma volume

Hematoma volume was measured at 24 and 72 h after ICH induction using hemoglobin assay as previously published [17]. The supernatant collected in the Evans Blue assay as described above was used [17]. The supernatant was mixed with Drabkin’s reagent (0.4 mL; Sigma Aldrich, MO) at a ratio of 1:4 at room temperature in the dark for 15 min. Optical density was measured at 540 nm with a spectrophotometer (GENESIS 10 S UV-Vis spectrophotometer; Thermo Fisher Scientific, MA) and quantified according to a standard curve, which showed the relationship between blood volume and total hemoglobin concentration in the brain tissue.

### Western blot

Protein expressions in the ipsilateral hemisphere were measured by western blot as previously described [3]. Protein samples with equal volumes were loaded on an SDS-PAGE gel, and then electrophoresed protein bands were transferred to a nitrocellulose membrane. The membrane was incubated with the following primary antibodies overnight at 4 °C: rabbit polyclonal anti-frizzled 7 (1:1000; ab64636, Abcam, MA), rabbit polyclonal anti-WISP1 (1:2000; ab178547, Abcam, MA), mouse monoclonal anti-Dvl (1:200; sc-166303, Santa Cruz Biotechnology, TX), rabbit monoclonal anti-β-Catenin (1:10,000; ab32572, Abcam, MA), rabbit polyclonal anti-phospho-β-Catenin (Ser33/37/Thr41) (1:1000; #9561, Cell Signaling, MA), rat monoclonal anti-ZO-1 (1:200, sc-33725, Santa Cruz Biotechnology, TX), rabbit polyclonal anti-Claudin-5 (1:500; ab15106, Abcam, MA), and mouse monoclonal anti-VE-Cadherin (1:500; sc-9989, Santa Cruz Biotechnology, TX). Appropriate secondary antibody (Santa Cruz Biotechnology, TX) was applied to the membranes and incubated for 1 h at room temperature. Immunoblots were probed and exposed to films. Band densities were analyzed using Image J software (NIH, Bethesda, MD).

### Immunohistochemistry

We performed double immunofluorescence staining as previously described [3]. The whole brains were fixed in 4% paraformaldehyde overnight followed by the dehydration in 20% sucrose solution and 30% sucrose solution. The brain specimens were stored − 20 °C until use. The brain specimen was cut into 10 μm thick coronal sections with a cryostat (CM1860; Leica Microsystems, Germany). Immunofluorescence staining was performed with the following primary antibodies: rabbit anti-frizzled 7 (1:100; ab64636, Abcam, MA), goat anti-frizzled 7 (1:100; # PA5-47232 Thermo Fisher, MA), goat anti-glial fibrillary acidic protein (GFAP) (1:200; ab53554, Abcam, MA), and mouse anti-neuronal nuclei (NeuN) (1:100; ab104224, Abcam, MA), and mouse anti-von Willebrand Factor (vWF) (1:100; sc-365712 Santa Cruz Biotechnology, TX. After the secondary antibody incubation, the brain sections were visualized and photographed with a fluorescence microscope (Leica Microsystems, Germany). Adobe Photoshop software was used to analyze the microphotographs.

### Perl’s staining

Brain samples were collected at 28 days after ICH and cut into coronal sections using a cryostat (CM1860; Leica Microsystems, Germany). Brain ferric iron content was measured with Perls’ Prussian blue staining as previously described [19]. Briefly, brain sections were treated with freshly prepared acid ferrocyanide solution for 20 min, washed well in distilled water and then counterstained with nuclear fast red. The area of iron-rich regions in microscopic images was quantified with Image J (NIH, Bethesda, MD).

### Statistical analysis

Mean and standard deviation were used to represent quantitative data. GraphPad Prism 6 was used for analysis. One-way ANOVA analysis followed by Tukey’s post hoc test for multiple-group comparisons was used to determine the differences in brain edema, neurological scores, Evans blue extravasation, hematoma volume and western blot test among all groups. The differences in rotarod and Morris water maze tests were analyzed by Two-way ANOVA with Bonferroni post hoc test. P value less than 0.05 was defined statistically significant.

## Results

### Mortality

The total mortality in ICH group was 1.1% (2/174) in this study. All mice in sham group survived. No significant difference was found in the mortality between the ICH groups (*p* > 0.05).

### ICH upregulated the brain expressions of FZD7 and WISP1

**Fig. 1:**
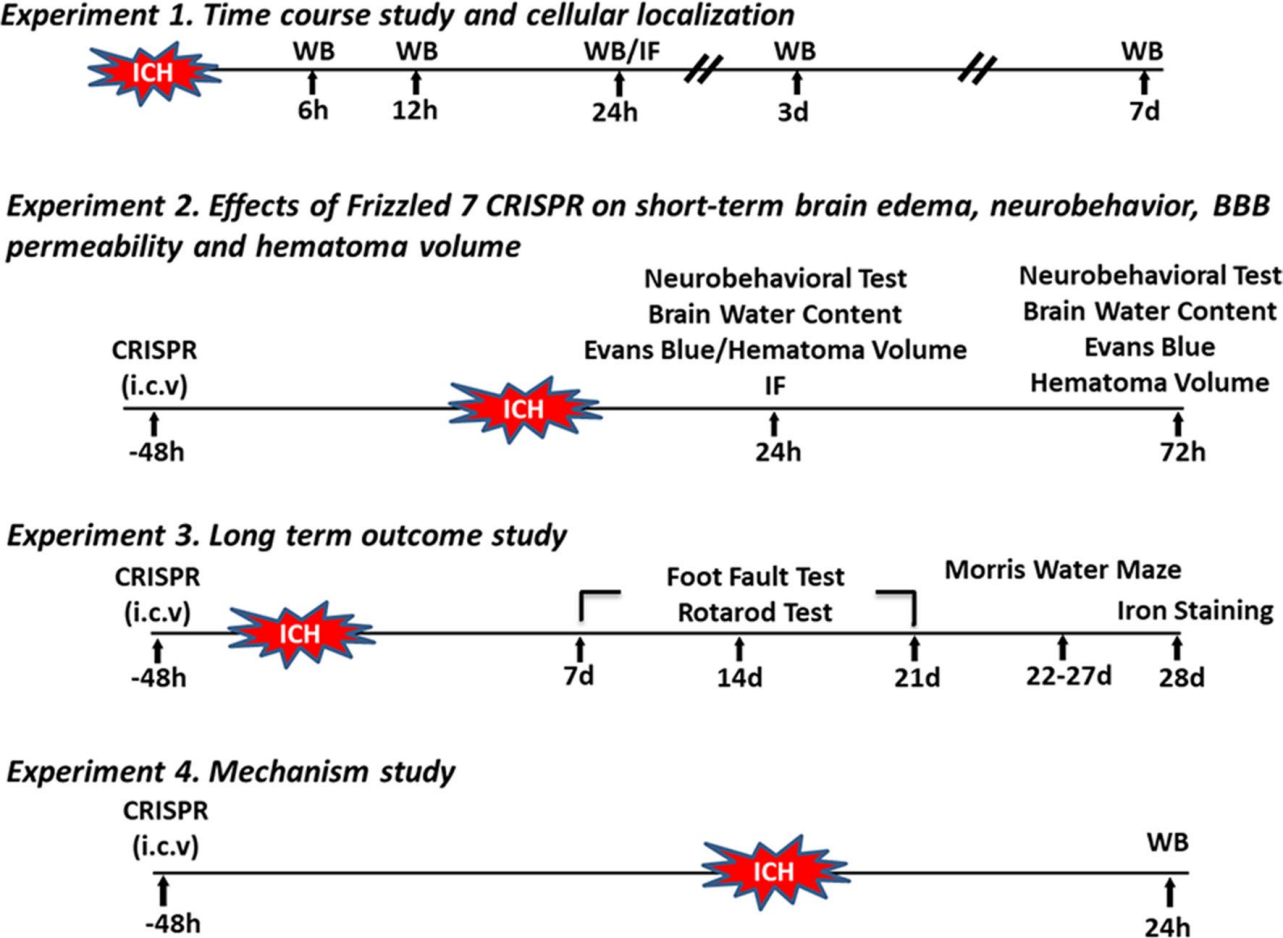
Experimental design. Experiment 1: Time course study and cellular localization. Experiment 2: Effects of Frizzled 7 CRISPR on short-term outcomes including neurobehavior, brain edema, BBB permeability and hematoma volume at 24 and 72 h after ICH. Experiment 3: Long term outcome study up to 28 days after ICH. Experiment 4: Mechanism study to elucidate the role of WISP1 as downstream mediator of FZD7. ICH: intracerebral hemorrhage; WB: Western blot; IF: immunofluorescence staining; CRISPR: Clustered Regularly Interspaced Palindromic Repeats; i.c.v.: intracerebroventricular

**Fig. 2:**
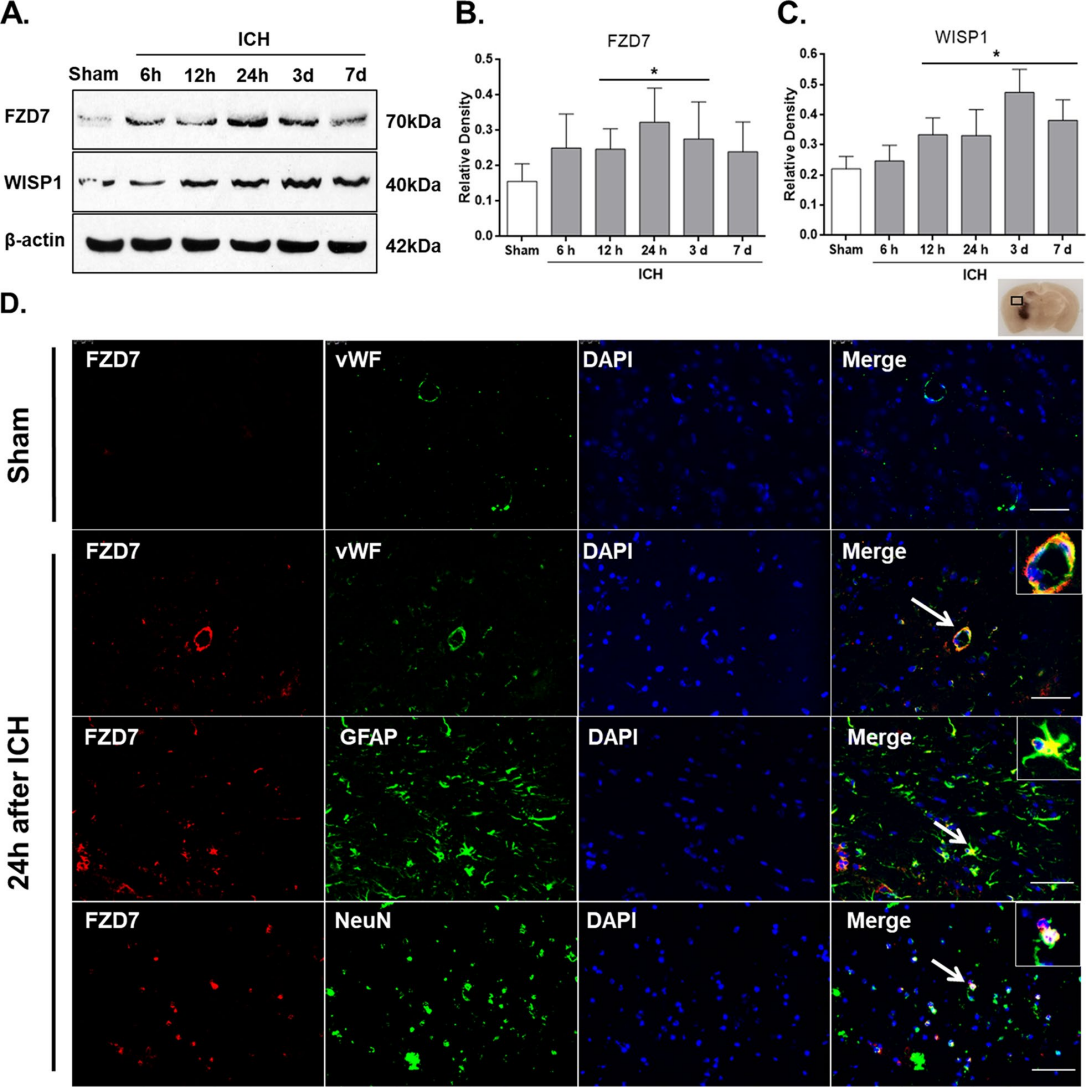
Temporal expressions of FZD7 and WISP1 along with cellular localization of FZD7 receptor after ICH. Representative western blot bands showing time course expressions of endogenous FZD7 and WISP1 (**A**) and densitometric quantification of FZD7 (**B**) and WISP1 (**C**) in the ipsilateral hemisphere after ICH. Data was represented as mean ± SD. FZD7 = Frizzled-7; WISP1: WNT1-inducible signaling pathway protein 1; ICH: intracerebral hemorrhage. n = 6 per group. *p < 0.05 vs. Sham group; One-way ANOVA, Tukey’s post hoc test. **D** Representative microphotographs of immunofluorescence co-staining of FZD7 (red) with endothelial cells (vWF, green), astrocytes (GFAP, green), and neurons (NeuN, green) at 24 h after ICH. Nuclei were stained with DAPI (blue). n = 6 per group. Scale bar = 50 μm

The protein levels of FZD7 and WISP1were measured in following groups: sham and ICH 6 h, 12 h, 24 h, 72 h and 7 days. The protein samples of sham group were extracted 24 h after the sham surgery (Fig. 1). Compared with sham group, the endogenous expressions of FZD7 in the ipsilateral hemisphere were increased at 12 h, 24 h and 3d post-ICH, and the endogenous expressions of WISP1 in the ipsilateral hemisphere were increased at 12 h, 24 h, 3 days and 7 days post-ICH (*p* < 0.05; Fig. 2A–C). The peak of FZD7 expression was observed at 24 h (Fig. 2B), while that of WISP1 was at 72 h post-ICH (Fig. 2C). Results of double immunofluorescence staining at 24 h post-ICH showed that FZD7 was co-localized with astrocytes, endothelial cells, and neurons at 24 h post-ICH, and it was mostly expressed in the area around the hematoma (Fig. 2D).

### Activation of FZD7 improved neurological function and decreased brain edema after ICH

**Fig. 3:**
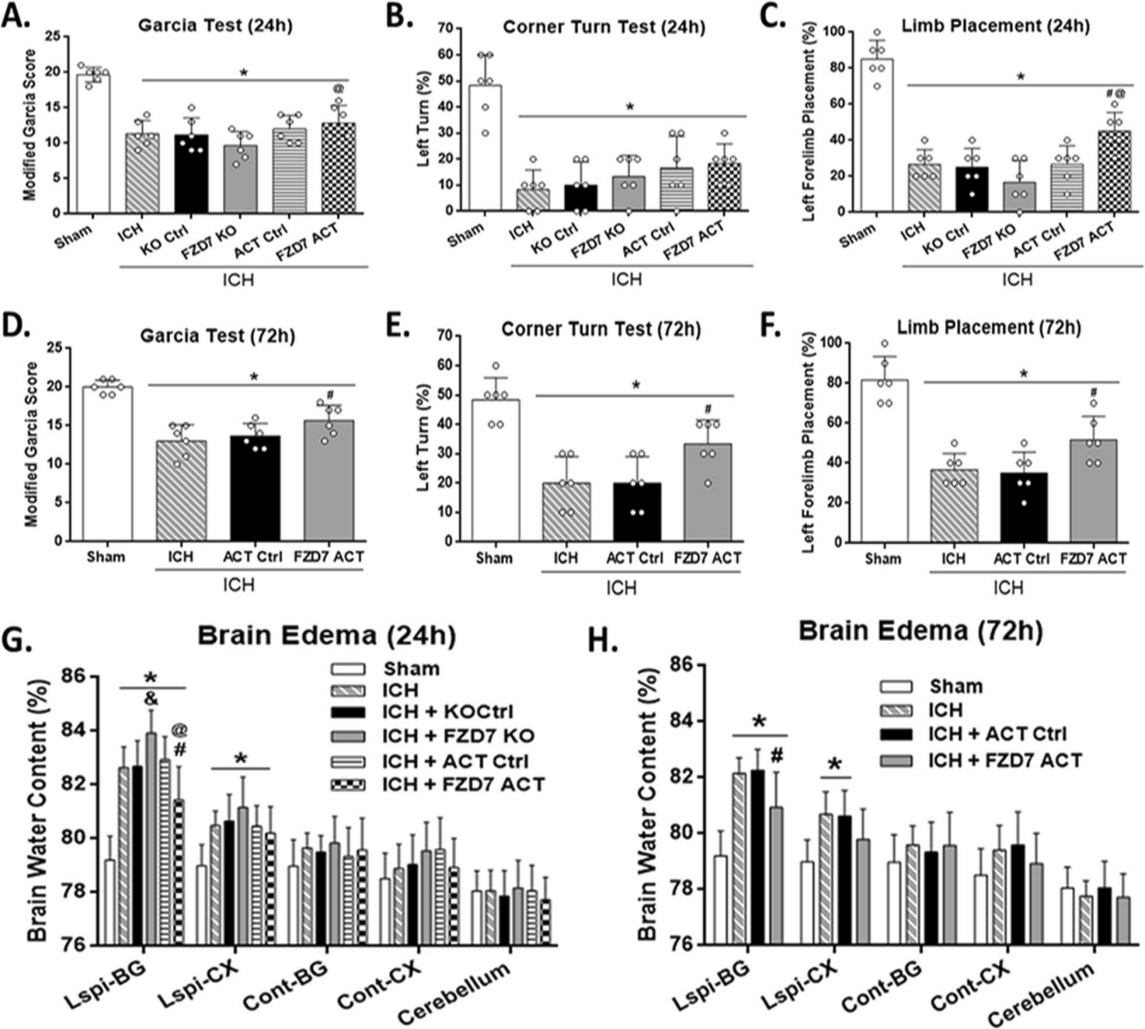
Effects of FZD7 CRISPR on neurobehavioral outcomes and brain edema after ICH. Modified Garcia test results of each group at 24 h (**A**) and 72 h (**D**) after ICH. Corner turn test results of each group at 24 h (**B**) and 72 h (**E**) after ICH. Limb placement test results of each group at 24 h (**C**) and 72 h (**F**) after ICH. Brain water content assessment at 24 h (**G**) and 72 h (**H**) after ICH. The bars represent the mean ± SD. FZD7: Frizzled-7; ICH: intracerebral hemorrhage; ACT Ctrl: activation control; KO Ctrl: knockdown control; FZD7 ACT: activation of frizzled-7; FZD7 KO: knockdown of frizzled-7; BG: basal ganglia; CX: cortex; Ipsi: ipsilateral; Cont: contralateral. n = 6 per group. *p < 0.05 vs. sham group; ^#^*p* < 0.05 vs. ICH + ACT Ctrl group; ^&^*p* < 0.05 vs. ICH + KO Ctrl group; ^@^*p* < 0.05 vs. ICH + FZD7 KO group; One-way ANOVA, Tukey’s post hoc test

To explore the effects of FZD7 on ICH outcomes, FZD7 activation or KO CRISPR was administered 48 h prior to ICH, and then neurobehavioral outcomes and brain edema were assessed at 24 and 72 h after ICH (Fig. 1). Intracerebroventricular administration of FDZ7 activation CRISPR significantly increased the endogenous expression of FZD7 in naive and ICH mice compared with the control CRISPR groups (see Additional file 1: Figure S1A, B). Correspondingly, intracerebroventricular administration of FDZ7 KO CRISPR significantly reduced the expression of FZD7 in naive and ICH mice compared with the control CRISPR groups (see Additional file 1: Figure S1A, C). These findings validated the activation and knockdown efficacies of FZD7 CRISPRs in this study. Compared with sham group, neurological score decreased and brain water content increased at 24 and 72 h after ICH in all the ICH groups, including ICH only, ICH + Ctrl (ACT and KO), ICH + FZD7 ACT, and ICH + FZD7 KO groups (*p* < 0.05; Fig. 3A–H). Activation of FZD7 reduced brain edema and improved limb placement test compared to the ICH control group (*p* < 0.05; Fig. 3G, C) and FZD7 activation improved brain edema and neurological scores in Garcia and limb placement tests compared with FZD7 knockdown group 24 h post-ICH (*p* < 0.05; Fig. 3G, A, C). The knockdown of FZD7 exacerbated brain edema (*p* < 0.05; Fig. 3G) and had a tendency to worsen neurological scores compared to control group 24 h post-ICH. Further studies were conducted to determine effects of FZD7 activation at 72 h post-ICH. Activation of FZD7 improved the neurological function (*p* < 0.05; Fig. 3D–F) and decreased brain edema (*p* < 0.05; Fig. 3H) in ipsilateral basal ganglia at 72 h after ICH.

### Activation of FZD7 reduced BBB permeability and hematoma volume after ICH

**Fig. 4:**
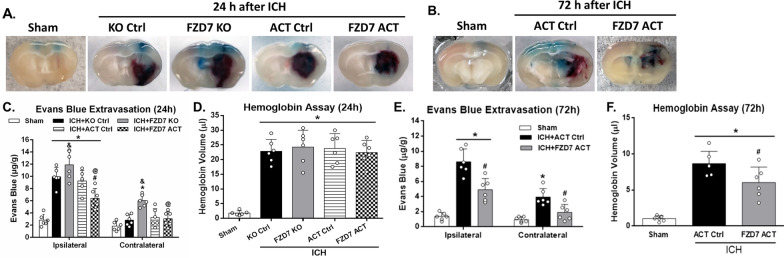
Effects of FZD7 CRISPR (activation or KO) on BBB permeability and hematoma volume. Evans blue extravasation at 24 h (**A**, **C**) and 72 h (**B**, **E**) after ICH; hemoglobin assay at 24 h (**D**) and 72 h (**F**) after ICH. The bars represent the mean ± SD. FZD7: Frizzled-7; ICH: intracerebral hemorrhage; ACT Ctrl: activation control; KO Ctrl: knockdown control; FZD7 ACT: activation of frizzled-7; FZD7 KO: knockdown of frizzled-7. n = 6 per group. *p < 0.05 vs. sham group; ^#^*p* < 0.05 vs. ICH + ACT Ctrl group; ^&^*p* < 0.05 vs. ICH + KO Ctrl group; ^@^*p* < 0.05 vs. ICH + FZD7 KO group; One-way ANOVA, Tukey’s post hoc test

BBB permeability was evaluated by Evans blue extravasation and hematoma volume was measured at 24 and 72 h after ICH (Fig. 1). Evans blue extravasation in the ipsilateral hemisphere significantly increased at 24 h after ICH and activation of FZD7 significantly decreased extravasation compared with control group, whereas knockdown of FZD7 increased Evans blue leakage in both hemispheres compared to control group (*p* < 0.05; Fig. 4A, C). Hemoglobin assay did not show significant difference in hematoma volumes at 24 h after ICH among all the ICH groups (*p* > 0.05; Fig. 4D). At 72 h after ICH, activation of FZD7 reduced both Evans blue leakage and hematoma volume compared with ICH + Ctrl group (*p* < 0.05; Fig. 4B, E, F).

### Activation of FZD7 improved long-term neurological function and reduced brain iron content after ICH

**Fig. 5:**
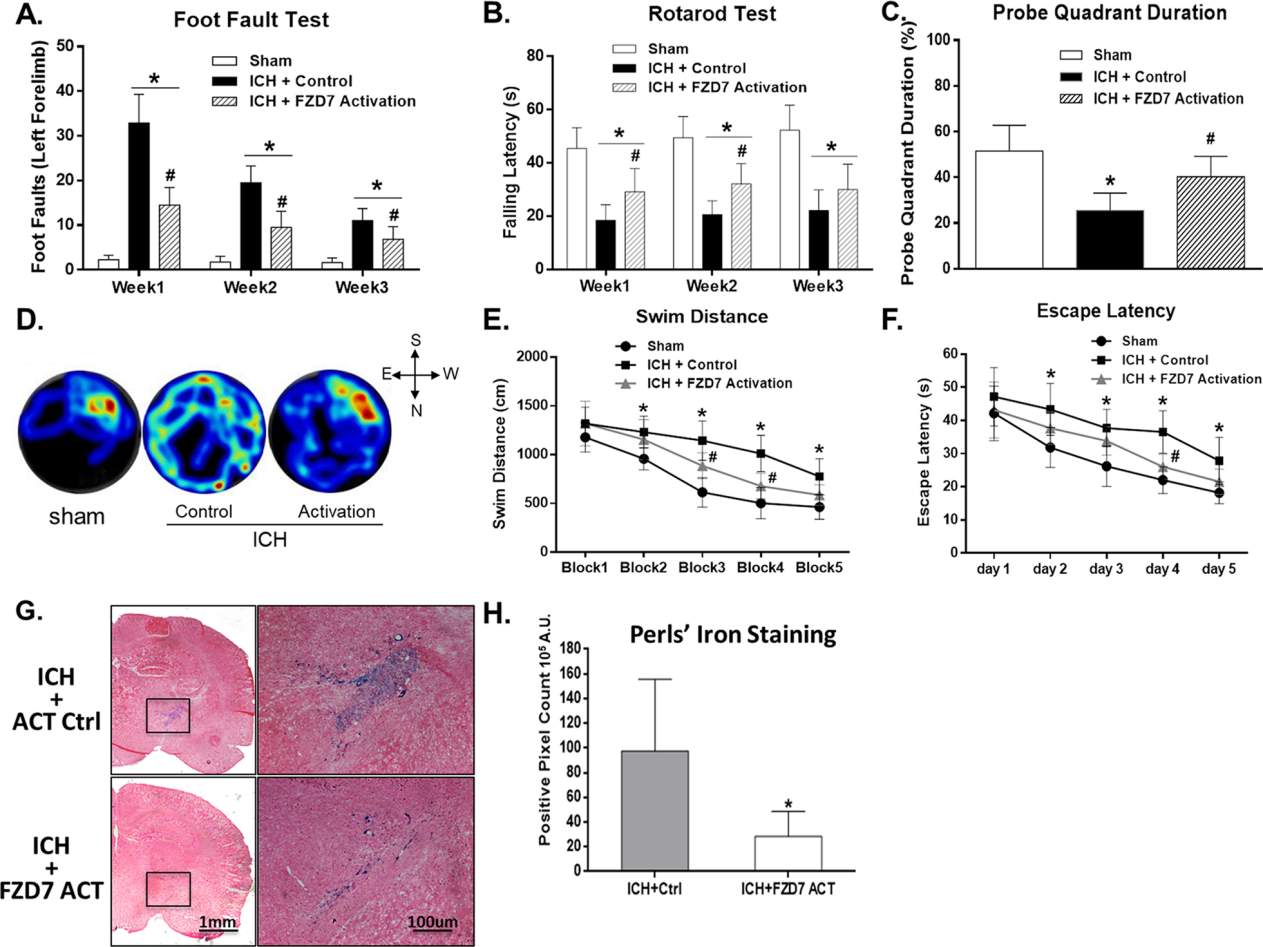
Effects of FZD7 activation on long-term neurobehavioral outcomes and iron deposition after ICH. Foot fault test (**A**) and rotarod test (**B**) on week 1, week 2, and week 3 after ICH. Quantification of probe quadrant duration in the probe trial of Morris water maze test on day 27 after ICH (**C**). Representative thermal imaging pictures of the probe trial (**D**). Swimming distance (**E**) and escape latency (**F**) of Morris water maze test on days 22 to 27 after ICH. Representative microphotographs (**G**, black rectangle in the left panel indicates the regions shown in the right panel under higher magnification) and quantification (**H**) of the Perl’s staining in the ipsilateral hemisphere. n = 6 per group. Data was represented as mean ± SD. FZD7: Frizzled-7; ICH: intracerebral hemorrhage. *p < 0.05 vs. Sham group; ^#^*p* < 0.05 vs. ICH + control group; Two-way repeated measures ANOVA, Tukey’s post hoc test (**A**, **B**, **E**, **F**), and One-way ANOVA, Tukey’s post hoc test (**C**, **H**)

We performed neurological tests including foot fault test and Rotarod test at days 7, 14, 21 after ICH (Fig. 1). Persistent neurologic dysfunction was observed by foot fault test and rotarod test in all ICH groups compared with the sham group (*p* < 0.05; Fig. 5A, B). Compared with the ICH + control group, FZD7 activation significantly reduced foot faults and improved falling latency from week 1 to week 3 after ICH (*p* < 0.05; Fig. 5A, B). Moreover, the results of the Morris water maze test conducted 22 to 27 days after ICH showed that compared with sham group, the ICH + control group spent less time in the probe quadrant, had increased swimming distance to find the platform, and longer escape latency suggesting that the learning ability and spatial memory in ICH mice were impaired (*p* < 0.05; Fig. 5C–F). The activation of FZD7 improved the memory and spatial learning compared with the ICH + control group as shown by shorter escape latency and swimming distance to platform, compared with the ICH + control group, as well as more time spent in probe quadrant (*p* < 0.05; Fig. 5C–F). Hemoglobin and iron release from the hematoma is a major contributor to ICH-induced brain injury [1, 20]. Iron staining and iron content was measured at 28 days after ICH induction which showed that iron content in the ipsilateral brain hemisphere was decreased in FZD7 activation group compared to the control group (*p* < 0.05; Fig. 5G, H).

### Activation of FZD7 attenuated BBB disruption via Dvl/β-Catenin/WISP1 signaling pathway

**Fig. 6:**
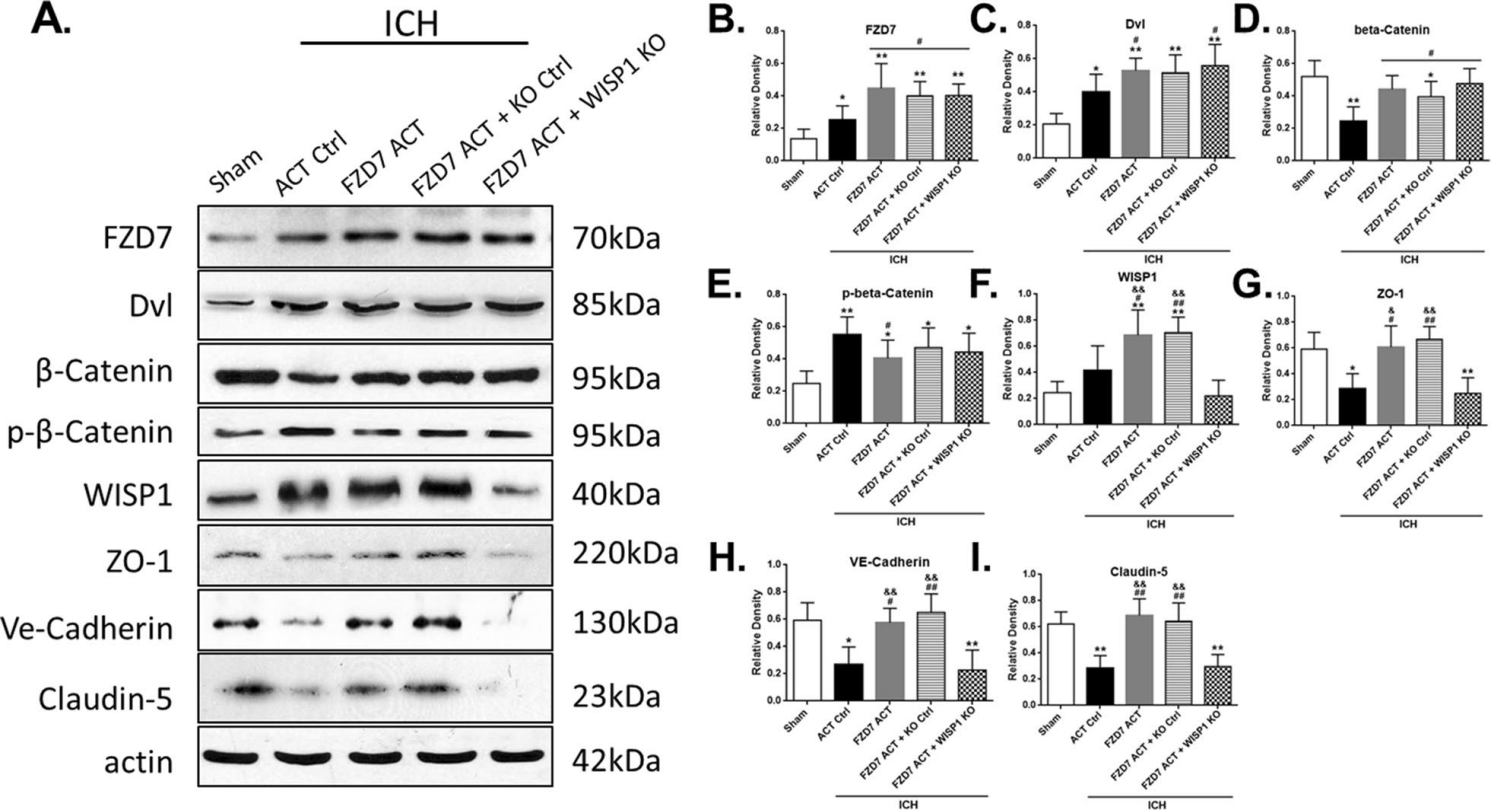
WISP1 knockdown abolished the effects of FZD7 activation on the expression of downstream TJPs. **A** Representative western blot bands at 24 h after ICH. **B**–**I** Densitometric quantification of FZD7, Dvl, β-Catenin, phospho-β-Catenin, WISP1, ZO-1, VE-Cadherin, and Claudin-5 in the ipsilateral hemisphere at 24 h after ICH. n = 6 per group; KO Ctrl: control CRISPR KO; FZD7 KO: Frizzled-7 CRISPR KO; ACT Ctrl: control CRISPR activation; WISP1 ACT: WISP1 CRISPR activation. Data was represented as mean ± SD. FZD7: Frizzled-7; WISP1: WNT1-inducible signaling pathway protein 1; TJPs: tight junction proteins; ICH: intracerebral hemorrhage. *p < 0.05 and **p < 0.01 vs. sham, ^#^*p* < 0.05 and ^##^*p* < 0.01 vs. ICH + ACT Ctrl, ^&^*p* < 0.05 and ^&&^*p* < 0.01 vs. ICH + FZD7 ACT + WISP1 KO; One-way ANOVA, Tukey’s post hoc test

**Fig. 7:**
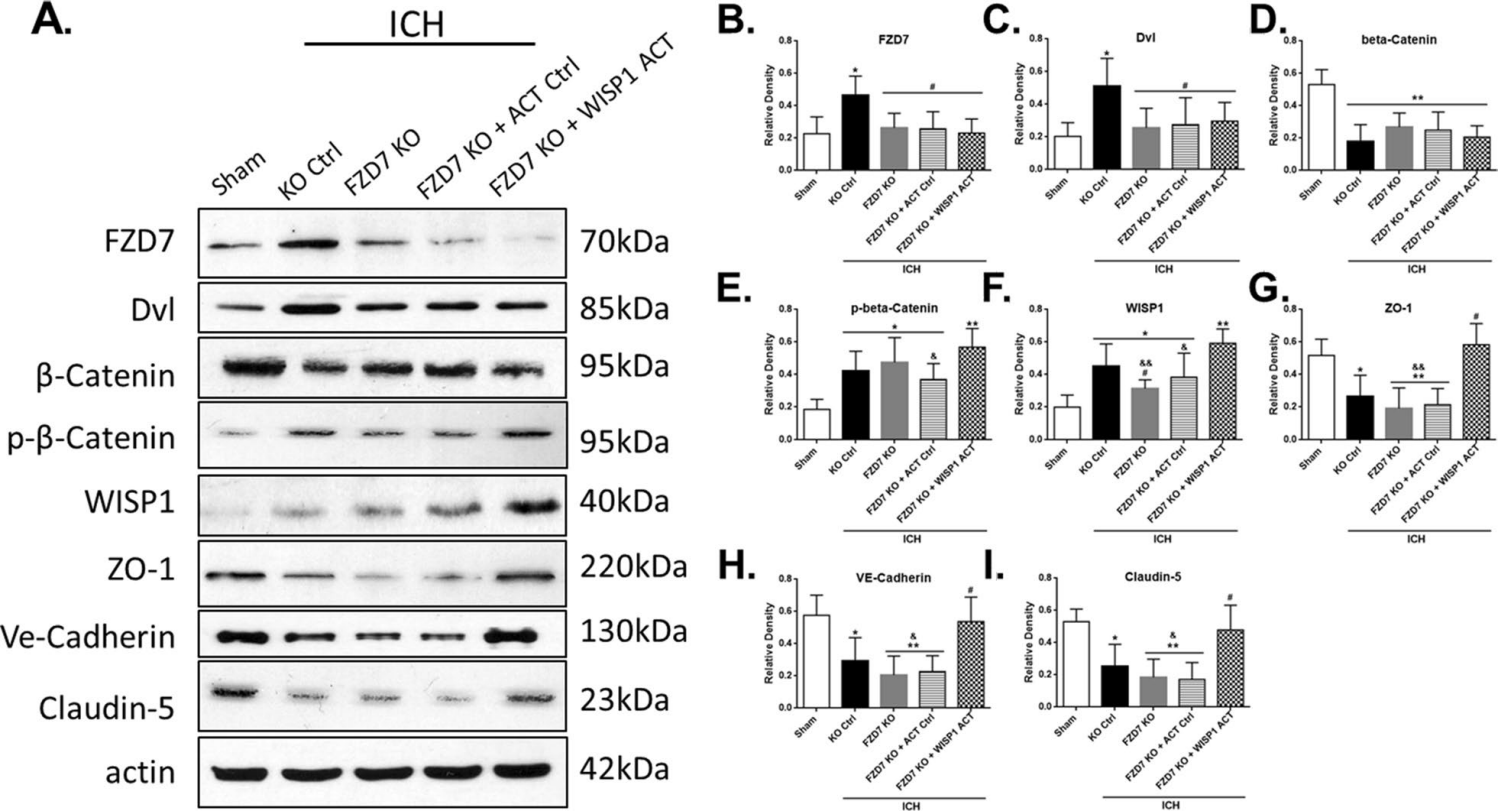
WISP1 activation abolished the effects of FZD7 knockdown on the expression of downstream TJPs. **A** Representative western blot bands at 24 h after ICH. **B**–**I** Densitometric quantification of FZD7, Dvl, β-Catenin, phospho-β-Catenin, WISP1, ZO-1, VE-Cadherin, and Claudin-5 in the ipsilateral hemisphere at 24 h after ICH. n = 6 per group; KO Ctrl: control CRISPR KO; FZD7 KO: Frizzled-7 CRISPR KO; ACT Ctrl: control CRISPR activation; WISP1 ACT: WISP1 CRISPR activation. Data was represented as mean ± SD. FZD7: Frizzled-7; WISP1: WNT1-inducible signaling pathway protein 1; TJPs: tight junction proteins; ICH: intracerebral hemorrhage. *p < 0.05 and **p < 0.01 vs. sham, ^#^*p* < 0.05 and ^##^*p* < 0.01 vs. ICH + ACT Ctrl, ^&^*p* < 0.05 and ^&&^*p* < 0.01 vs. ICH + FZD7 ACT + WISP1 KO; One-way ANOVA, Tukey’s post hoc test

The expression of FZD7 and its downstream signaling molecules was evaluated by Western blots at 24 h after ICH (Fig. 1). To verify the proposed pathway, FZD7 activation CRISPR and WISP1 KO CRISPR was administered. FZD7 activation CRISPR increased the expression of FZD7, and the downstream mediators Dvl, β-Catenin, WISP1 in ipsilateral hemisphere at 24 h post-ICH and inhibited phospho-β-Catenin expression. It resulted in a higher expressions of BBB endothelial adherens junction VE-Cadherin and TJs including ZO-1 and Claudin-5 compared to ICH + control group (*p* < 0.05; Fig. 6A–I). WISP1 knockdown did not affect the upstream factors but reversed the effects of FZD7 activation on the expressions of BBB junction proteins compared with ICH + FZD7 ACT group (*p* < 0.05; Fig. 6A–I). To further verify the proposed pathway, FZD7 KO CRISPR and WISP1 activation CRISPR was administered. Knockdown of FZD7 decreased the expressions of FZD7, Dvl, β-Catenin, WISP1 in the ipsilateral hemisphere at 24 h post-ICH and increased phospho-β-Catenin expression, which inhibited the expression of VE-Cadherin, Claudin-5, and ZO-1, compared with ICH + control group (*p* < 0.05; Fig. 7A–I). Activation of WISP1 abolished the effects of FZD7 knockdown on BBB junction proteins expressions compared with ICH + FZD7 KO group at 24 h after ICH (*p* < 0.05; Fig. 7A–I). Additionally, the efficacy of WISP1 CRISPR activation or knockdown was verified in naive and ICH mice. Intracerebroventricular administration of WISP1 activation or KO CRISPR significantly increased or reduced the endogenous expression of WISP1 compared with the control CRISPR group in both naive and ICH mice (Additional file 2: Figure S2A–C). BBB permeability was evaluated by Evans blue extravasation at 72 h after ICH to show the effects of WISP1 knockdown or activation on the BBB functional integrity (Additional file 3). WISP1 expression peaked at 72 h after ICH (p < 0.05; Fig. 2A, C). Evans blue extravasation in the ipsilateral hemisphere significantly increased at 72 h after ICH and activation of WISP1 significantly decreased extravasation compared with control group, whereas knockdown of WISP1 increased Evans blue leakage in both hemispheres compared to control group (p < 0.05; Additional file 3).

## Discussion

In this study, we evaluated the role of FZD7 signaling pathway in BBB permeability after ICH. Using a collagenase-induced mouse model of ICH, we found that: (1) The endogenous expressions of FZD7 and the downstream protein WISP1 were significantly increased in the ipsilateral hemisphere following ICH, and their expression peaked at 24 and 72 h after ICH, respectively; (2) FZD7 CRISPR activation attenuated brain edema and BBB disruption as well as improved neurological deficits following ICH; (3) FZD7 CRISPR KO aggravated brain edema, BBB permeability and neurological deficits after ICH; (4) FZD7 CRISPR activation increased the expressions of Dvl, β-Catenin, WISP1, ZO-1, Claudin-5, and VE-Cadherin, as well as inhibited the expression of phospho-β-Catenin in the ipsilateral brain hemisphere at 24 h post-ICH; (5) WISP1 CRISPR KO abolished the up-regulation of TJ proteins expressions by FZD7 CRISPR activation. On the other hand, WISP1 CRISPR activation reversed the suppression of TJ proteins expression by FZD7 CRISPR KO. Collectively, these results indicated that the activation of FZD7 attenuated BBB disruption and improved neurological deficits after ICH, which was at least in part mediated by Dvl/β-Catenin/WISP1 signaling pathway.

BBB integrity is critical for brain homeostasis [21, 22], and its integrity is maintained by the endothelial cells (ECs) [23]. BBB disruption is not only a common pathophysiology of various neurological diseases, but also promotes the onset of these diseases [24–27]. In the setting of ICH, BBB destruction is a vital mechanism of secondary brain injury [3]. Extensive efforts have been made to better understand the physiological development and maintenance of BBB as well as to identify the potential targets for BBB repair under pathological conditions [3, 4, 16, 20, 28].

Canonical Wnt/β-catenin signaling pathway induces the formation and maturation of BBB during individual development [6]. Dephosphorylated β-catenin is its active form, which can trigger the downstream β-catenin canonical Wnt pathway [6, 12]. However, its importance has not been stressed for normal BBB function in adults due to the rarely detectable β-catenin in the EC nucleus of adult cerebral vessels [6]. Recent studies have found that Wnt/β-catenin pathway is essential for BBB integrity and CNS homeostasis after CNS diseases such as ischemic stroke, hemorrhagic stroke and epilepsy [29–31]. The expression of nuclear β-catenin and TJ proteins claudin-3 were decreased in endothelial cells of brain lesions in patients with hemorrhagic stroke [29]. This implies that the β-catenin signaling may serve as a protective factor against BBB disruption following ICH.

As a new receptor expressed on the surface of ECs, FZD7 recruits Dvl at cell membranes in EC to activate both the Wnt canonical β-catenin pathway and the noncanonical Wnt pathway [32–34]. FZD7 has been shown to control vascular permeability mainly through the activating of Dvl/β-catenin canonical Wnt pathway [10]. Previous study demonstrated that angiogenesis was promoted by FZD7 via the Wnt canonical pathway. This FZD7 signaling occurs mainly by activating the Dvl/β-catenin canonical Wnt signaling [10].

We studied the effects of FZD7 receptor-mediated Wnt/β-Catenin signaling pathway and its downstream target protein WISP1 on BBB preservation in mice after ICH. We evaluated the endogenous expression of FZD7 and WISP1 in brain samples at various time-points after ICH in mice. Brain RNA-Seq database (and Single-cell RNA-Seq database show that FZD7 is expressed in mouse brain endothelial cells, but the expression level was relatively low compared to other cell types. Similarly, our data showed that the expression level of FZD7 in the sham groups was very low. But interestingly, ICH injury resulted in an increase in endogenous FZD7 expression with peak at 24 h after ICH, accompanied with a time-dependent up-regulation of WISP1 expression that peaked at 72 h after ICH. We found that FZD7 was expressed in ECs, astrocytes and neuronal cells 24 h after ICH. This temporal pattern implicated the activation of FZD/WISP1 may be an endogenous protection mechanism against neurological dysfunction caused by ICH, although the extent of protection was not enough to completely offset the damage.

Using the commercially available CRISPR to activate or inhibit FZD7 receptor, we verified the protective role the FZD7 receptor in ICH. CRISPR has emerged as a powerful and broadly accessible tool for unbiased forward gain-of-function and loss-of-function gene in mammalian cells [35]. We found that the activation of FZD7 with activation CRISPR reduced cerebral edema and improved short-term or long-term neurological deficits in mice after ICH. These benefits were possibly associated with reduced BBB permeability and BBB junction protein expression preservation within the ipsilateral brain tissues. On the other hand, FZD7 CRISPR KO showed the opposite effects as elicited by aggravated BBB permeability, cerebral edema, and worsened neurological deficits following ICH.

The continuous intercellular TJs make the BBB ECs unique from other ECs [23], which stabilizes the tight connection and regulates permeability of the BBB. During pathophysiological conditions, the activated endothelium changes the endothelial cytoskeleton in BBB [36]. VE-Cadherin is an endothelial-specific intercellular adhesion protein in adherens junctions and plays a key role in the permeability of endothelial cells [37]. In various brain injury models, the permeability of BBB to Evans blue staining was found to increase when the expression of VE-cadherin decreased [38]. VE-cadherin controls the expression of the TJ protein claudin-5 [39], which is known to be degraded after ICH [28]. ZO-1 is another TJ protein that is critical to maintaining BBB integrity. We evaluated the expression of BBB junction proteins including VE-Cadherin, Claudin-5 and ZO-1 in the present study. The expressions of VE-cadherin, Claudin-5 and ZO-1 were decreased in the ipsilateral brain tissues at 24 h after ICH, which were significantly rescued by FZD7 activation but exacerbated by FZD7 CRISPR KO.

Interestingly, despite the insignificant difference at 24 h after ICH, we found that the hematoma volumes were significantly smaller in FZD7 CRISPR activation treated ICH mice compared to control CRISPR ICH group at 72 h after ICH. Likewise, brain tissue iron staining at 28 days post-ICH was also significantly less in FZD7 CRISPR activation group. We speculated that early FZD7 activation may facilitate beneficial angiogenesis process [10] and thereby promote hematoma resolution. Nevertheless, the effect of FZD7 activation on post-ICH hematoma resolution needs to be further investigated.

We evaluated the downstream mediators activated by FZD7 that may regulate the integrity of TJs between endothelial cells after ICH. The activation of FZD7 causes Dv1 to accumulate on the cell membrane, which is essential for initiating the downstream β-Catenin signaling cascade. The transcription factor β-catenin is central to the Wnt signaling pathway that maintains the stability of endothelial barrier and integrity of adult BBB by controlling TJ protein transcription in brain ECs [30, 40]. Previous studies showed that one of pathogenic factors of neurological diseases such as hemorrhagic stroke, epilepsy and central nervous system inflammation is BBB dysfunction caused by defective β-catenin transcription activity [30]. Inactivation of β-catenin can lead to the destruction of BBB integrity in adult mice [30]. WISP1 is the downstream target protein of β-catenin [41], which promotes angiogenesis in cancers [42–44] and cell survival in neurological pathologies [45, 46]. WISP1 knockout in different melanoma cells was found to down-regulate N-cadherin and fibronectin [13]. In human osteosarcoma, WISP1 actively regulates angiogenesis by controlling the expression of VEGF-A [47]. WISP1 was increased after myocardial infarction and contributed to the beneficial cardiac-specific angiogenesis via directly regulating human cardiac-specific ECs [41]. We observed that increase in WISP1 expression accompanied the up-regulation of FZD7 expression after ICH in mice. To elucidate the connection between WISP1 and FZD7 and the effect on vascular permeability, we used both FZD7 and WISP1 CRISPRs. In ICH mice with FZD7 CRISPR activation, the administration of WISP1 CRISPR KO did not change expressions of upstream mediators Dvl and β-Catenin but it down-regulated the expressions of downstream BBB junction proteins VE-Cadherin, Claudin-5 and ZO-1. Conversely, when we knocked down FZD7 but up-regulated WISP1 in ICH mice, the expressions of Dvl and β-Catenin remained unchanged but the expressions of BBB junction proteins were preserved compared with control ICH mice. Further study was performed to verify the effect of WISP1 knockdown or activation on the functional integrity of the BBB. We found that activating WISP1 reduced the permeability of the blood–brain barrier, while knocking down WISP1 showed the opposite effect. These data strengthened our hypothesis that WISP1 might be a key mediator of FZD7 signaling in brain microvascular endothelial cells. The above research results suggested that WISP1 is a potential downstream mediator of FZD7/Dvl/β-Catenin signaling pathway and regulates BBB permeability by up regulating the expressions of BBB junction proteins after ICH in mice.

### Limitations

There are some limitations in our study. First, we did not explore the mechanism underlying the endogenous up-regulation of FZD7 expression after ICH injury. Second, our focus was endothelial FZD7 signaling and BBB preservation. However, we did not exclude the role of other possible neuroprotective mechanisms involving astrocytes, neurons and/or pericytes that may also contribute to neurovascular injury after ICH. Third, the effects of FZD7 signaling on angiogenesis and hematoma resolution after ICH needs to be investigated in future studies. Lastly, we used Evans blue extravasation to evaluate BBB permeability which only demonstrates the leakage of large molecular weight substances such as albumin. We did not evaluate leakage of small molecular weight substances, and therefore the extent of BBB leakiness was not determined in this study.

## Conclusions

In conclusion, our study suggests that the activation of FZD7 alleviated BBB disruption, neurological impairments and brain edema in mice after ICH. This neuroprotection was at least in part mediated via up-regulating Dvl/β-Catenin/WISP1 signaling pathway. Therefore, FZD7 may be a potential therapeutic target for BBB protection and improvement of outcomes in ICH patients.

## Supplementary Information

**Additional file 1: Figure S1. **Efficacy of FZD7 CRISPR activation or knockdown in naive and ICH mice. (A) Representative western blot bands at 24 h after ICH. (B-C) Densitometric quantification of FZD7. n = 4 per group; Ctrl CRISPR = control CRISPR; CRISPR ACT = Frizzled-7 CRISPR activation; CRISPR KO = Frizzled-7 CRISPR KO. Data was represented as mean ± SD. FZD7 = Frizzled-7; ICH = intracerebral hemorrhage. *p < 0.05 vs. Naive + Control CRISPR, @*p* < 0.05 vs. ICH + Control CRISPR; One-way ANOVA, Tukey’s post hoc test.

**Additional file 2: Figure S2.** Efficacy of WISP1 CRISPR activation or knockdown in naive and ICH mice. (A) Representative western blot bands at 24 h after ICH. (B-C) Densitometric quantification of WISP1. n = 4 per group; Ctrl CRISPR = control CRISPR; CRISPR ACT = WISP1 CRISPR activation; CRISPR KO = WISP1 CRISPR KO. Data was represented as mean ± SD. ICH = intracerebral hemorrhage, WISP1 = WNT1-inducible signaling pathway protein 1. *p < 0.05 vs. Naive + Control CRISPR, @*p* < 0.05 vs. ICH + Control CRISPR; One-way ANOVA, Tukey’s post hoc test.

**Additional file 3: Figure S3.** Effects of WISP1 CRISPR (activation or KO) on BBB permeability. Evans blue extravasation at 72 h (A, B) after ICH. The bars represent the mean ± SD. ICH = intracerebral hemorrhage; WISP1 = WNT1-inducible signaling pathway protein 1; ACT Ctrl = activation control; KO Ctrl = knockdown control; WISP1 ACT = activation of WISP1; WISP1 KO = knockdown of WISP1. n = 4 per group. *p < 0.05 vs. sham group; #*p* < 0.05 vs. ICH + ACT Ctrl group; &*p* < 0.05 vs. ICH + KO Ctrl group; @*p* < 0.05 vs. ICH + WISP1 KO group; One-way ANOVA, Tukey’s post hoc test.

## Data Availability

Data are available from the corresponding author with reasonable request.

## Abbreviations

BBB: Blood–brain barrier
ICH: Intracerebral hemorrhage
FZD7: Frizzled-7
WISP1: WNT1-inducible signaling pathway protein 1
ACT: Activation
KO: Knockdown
TJPs: Tight junction proteins
Dvl: Disheveled
ZO-1: Zona occludens-1
CRISPR: Clustered Regularly Interspaced Palindromic Repeats
Ctrl: Control
WW: Wet weight
DW: Dry weight
PBS: Phosphate buffered saline
TCA: Trichloroacetic acid
GFAP: Glial fibrillary acidic protein
NeuN: Neuronal nuclei
vWF: Von Willebrand Factor
ECs: Endothelial cells
