# Supplementary figures and images for: Activation of Frizzled-7 attenuates blood–brain barrier disruption through Dvl/β-catenin/WISP1 signaling pathway after intracerebral hemorrhage in mice
Source: Fluids Barriers CNS. 2021 Sep 26;18:44. doi: 10.1186/s12987-021-00278-9 (PMC8474841; doi:10.1186/s12987-021-00278-9)

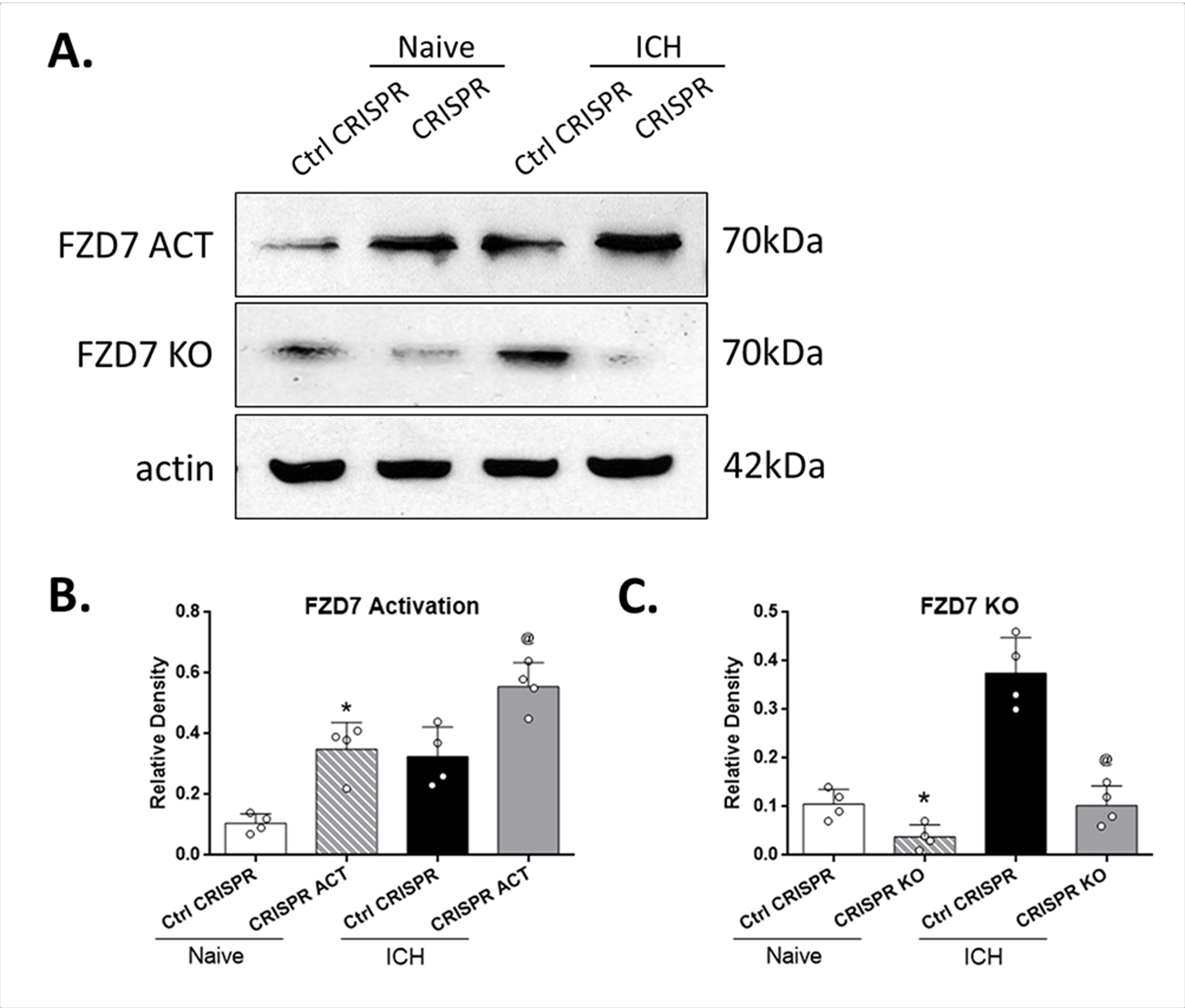

Supplement: Supplementary file 1 — Additional file 1: Figure S1. Efficacy of FZD7 CRISPR activation or knockdown in naive and ICH mice. (A) Representative western blot bands at 24 h after ICH. (B-C) Densitometric quantification of FZD7. n = 4 per group; Ctrl CRISPR = control CRISPR; CRISPR ACT = Frizzled-7 CRISPR activation; CRISPR KO = Frizzled-7 CRISPR KO. Data was represented as mean ± SD. FZD7 = Frizzled-7; ICH = intracerebral hemorrhage. *p < 0.05 vs. Naive + Control CRISPR, @p < 0.05 vs. ICH + Control CRISPR; One-way ANOVA, Tukey’s post hoc test. [file 12987_2021_278_MOESM1_ESM.tif]

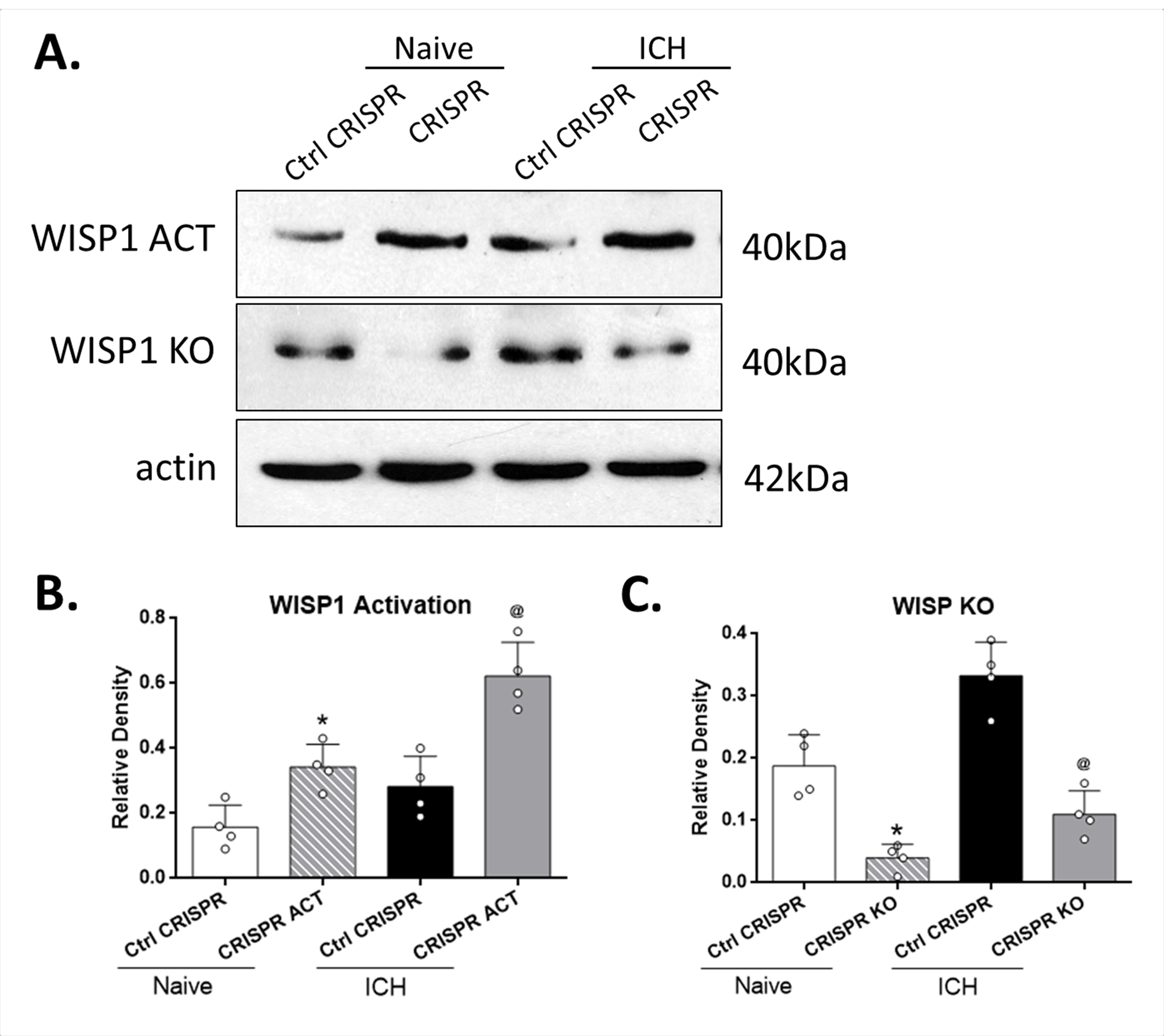

Supplement: Supplementary file 2 — Additional file 2: Figure S2. Efficacy of WISP1 CRISPR activation or knockdown in naive and ICH mice. (A) Representative western blot bands at 24 h after ICH. (B-C) Densitometric quantification of WISP1. n = 4 per group; Ctrl CRISPR = control CRISPR; CRISPR ACT = WISP1 CRISPR activation; CRISPR KO = WISP1 CRISPR KO. Data was represented as mean ± SD. ICH = intracerebral hemorrhage, WISP1 = WNT1-inducible signaling pathway protein 1. *p < 0.05 vs. Naive + Control CRISPR, @p < 0.05 vs. ICH + Control CRISPR; One-way ANOVA, Tukey’s post hoc test. [file 12987_2021_278_MOESM2_ESM.tif]

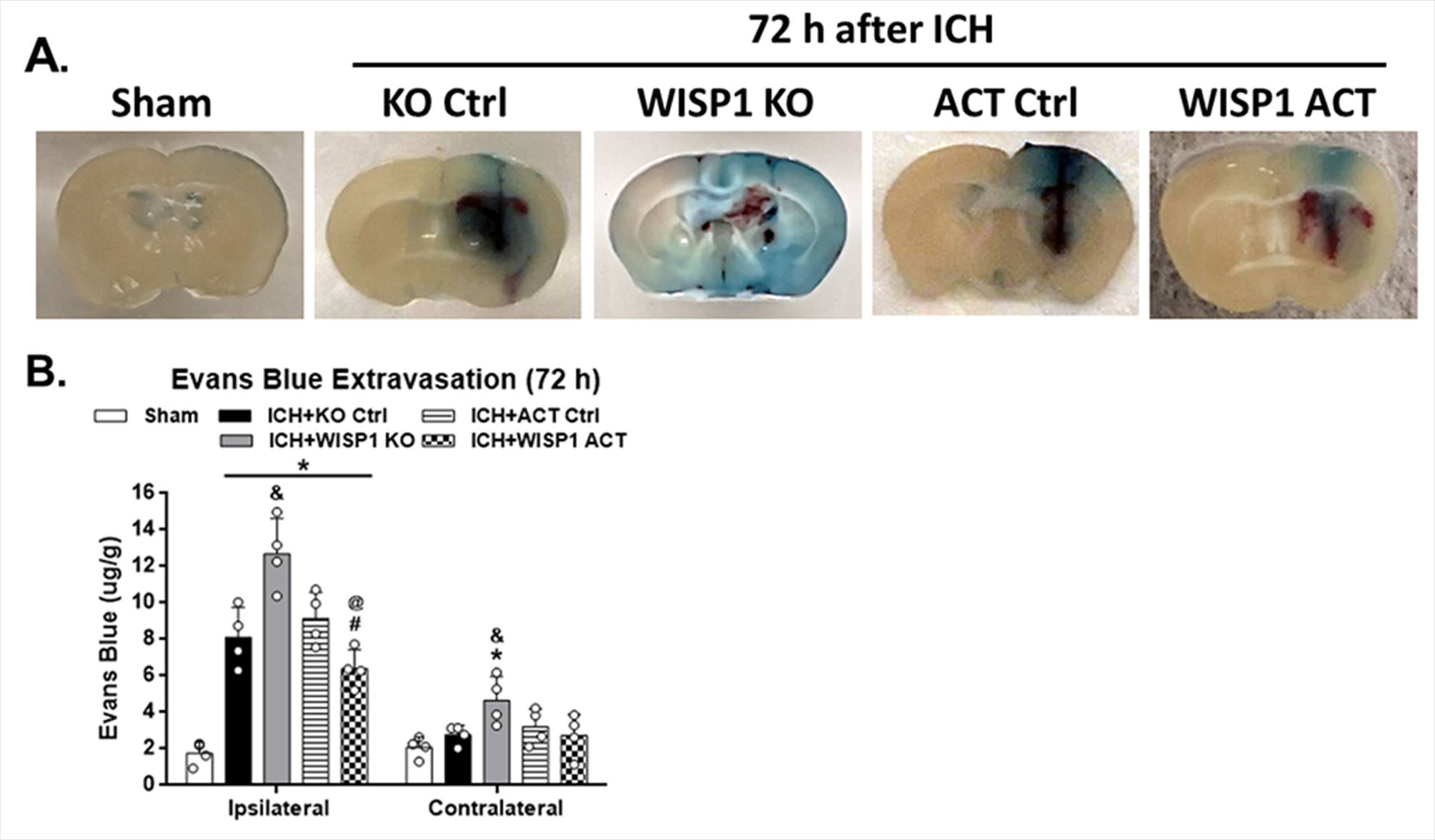

Supplement: Supplementary file 3 — Additional file 3: Figure S3. Effects of WISP1 CRISPR (activation or KO) on BBB permeability. Evans blue extravasation at 72 h (A, B) after ICH. The bars represent the mean ± SD. ICH = intracerebral hemorrhage; WISP1 = WNT1-inducible signaling pathway protein 1; ACT Ctrl = activation control; KO Ctrl = knockdown control; WISP1 ACT = activation of WISP1; WISP1 KO = knockdown of WISP1. n = 4 per group. *p < 0.05 vs. sham group; #p < 0.05 vs. ICH + ACT Ctrl group; &p < 0.05 vs. ICH + KO Ctrl group; @p < 0.05 vs. ICH + WISP1 KO group; One-way ANOVA, Tukey’s post hoc test. [file 12987_2021_278_MOESM3_ESM.tif]
